# [^18^F]fluoroethylcholine-PET/CT imaging for radiation treatment planning of recurrent and primary prostate cancer with dose escalation to PET/CT-positive lymph nodes

**DOI:** 10.1186/1748-717X-6-44

**Published:** 2011-05-01

**Authors:** Florian Würschmidt, Cordula Petersen, Andreas Wahl, Jörg Dahle, Matthias Kretschmer

**Affiliations:** 1 Radiologische Allianz Hamburg, D-22767 Hamburg, Germany; 2 Klinik für Strahlentherapie & Radioonkologie, UKE, Hamburg, Germany

## Abstract

**Background:**

At present there is no consensus on irradiation treatment volumes for intermediate to high-risk primary cancers or recurrent disease. Conventional imaging modalities, such as CT, MRI and transrectal ultrasound, are considered suboptimal for treatment decisions. Choline-PET/CT might be considered as the imaging modality in radiooncology to select and delineate clinical target volumes extending the prostate gland or prostate fossa. In conjunction with intensity modulated radiotherapy (IMRT) and imaged guided radiotherapy (IGRT), it might offer the opportunity of dose escalation to selected sites while avoiding unnecessary irradiation of healthy tissues.

**Methods:**

Twenty-six patients with primary (n = 7) or recurrent (n = 19) prostate cancer received Choline-PET/CT planned 3D conformal or intensity modulated radiotherapy. The median age of the patients was 65 yrs (range 45 to 78 yrs). PET/CT-scans with F18-fluoroethylcholine (FEC) were performed on a combined PET/CT-scanner equipped for radiation therapy planning.

The majority of patients had intermediate to high risk prostate cancer. All patients received 3D conformal or intensity modulated and imaged guided radiotherapy with megavoltage cone beam CT. The median dose to primary tumours was 75.6 Gy and to FEC-positive recurrent lymph nodal sites 66,6 Gy. The median follow-up time was 28.8 months.

**Results:**

The mean SUV_max _in primary cancer was 5,97 in the prostate gland and 3,2 in pelvic lymph nodes. Patients with recurrent cancer had a mean SUV_max _of 4,38. Two patients had negative PET/CT scans. At 28 months the overall survival rate is 94%. Biochemical relapse free survival is 83% for primary cancer and 49% for recurrent tumours. Distant disease free survival is 100% and 75% for primary and recurrent cancer, respectively. Acute normal tissue toxicity was mild in 85% and moderate (grade 2) in 15%. No or mild late side effects were observed in the majority of patients (84%). One patient had a severe bladder shrinkage (grade 4) after a previous treatment with TUR of the prostate and seed implantation.

**Conclusions:**

FEC-PET/CT planning could be helpful in dose escalation to lymph nodal sites of prostate cancer.

## Background

In primary and recurrent prostate cancer, the diagnostic accuracy of conventional imaging modalities, such as transrectal ultrasound, computed tomography (CT) and magnetic resonance (MR) imaging, is still considered suboptimal in the management of these patients [1]. A substantial number of patients fail within 10 years after either radical prostatectomy or radiotherapy and precise information about the site of recurrence is crucial for the choice of an adequate therapeutic strategy [2]. At present there is no consensus on irradiation treatment volumes of intermediate to high-risk primary cancers or recurrent disease. In recurrent cancer, most frequently the prostatic fossa with or without the seminal vesicles but not pelvic lymph nodes have been recommended as clinical target volumes [3,4]. The potential roles of PET/CT in radiooncology are (1) patient selection for treatment and (2) target volume selection and delineation, because PET/CT with radiolabelled choline (C11-choline, F18-fluoroethylcholine, F18-fluoromethycholine) presents high values of sensitivity and specificity in visualizing sites of disease especially at the lymph nodal level [5,6], though, its value in primary cancer is a matter of debate. Thus, Choline-PET/CT might be considered as the imaging modality in radiooncology to select and delineate clinical target volumes extending the prostate gland or prostate fossa (with seminal vesicles) [2]. In conjunction with high-precision radiation therapy techniques, i.e., intensity modulated radiotherapy (IMRT) and imaged guided radiotherapy (IGRT), it might offer the opportunity of dose escalation to selected sites and better tumour control while avoiding unnecessary inclusion of normal healthy tissues.

Here we report on prostate cancer patients with intermediate to high-risk primary or recurrent disease, who underwent Choline-PET/CT planned 3D conformal or IMRT and IGRT radiotherapy with dose escalation to PET/CT-positive disease sites.

## Methods

### Patients

Between November 2006 and July 2010, twenty-six patients received F18-fluorethylcholine-PET/CT as part of the staging procedure and for radiation therapy treatment planning. Nineteen patients presented with a recurrent prostate cancer (biochemical relapse) after previous radical prostatectomy with or without lymphadenectomy of which one patient presented with PET/CT positive lymph nodes (iliac and paraaortal) within two years. Seven patients had primary disease.

Details of patient and tumour characteristics are given in Table 1. The median age of the patients was 65 yrs (range 45 to 78 yrs). The majority of recurrent prostate cancer patients had intermediate to high risk cancer with 10/19 with initial pT3/4 disease and Gleason score of 7 or higher (13/19). The primary tumours were cT3 in 5/7 and Gleason score 7 or higher in 5/6 (1: not available). The median iPSA was 10,4 ng/ml (range: 2,5 to 731) in primary cancer and 12,1 ng/ml (range: 3,35 to 43) in recurrent cancer. The median PSA at time of FEC-PET/CT in primary cancer was 10,4 ng/ml (range 0,2 to 115) and 1,9 ng/ml (range 0,42 to 65) in recurrent disease.

**Table 1:** Patient and tumor characteristics

**Pt**.	Age (yr)	stage	Gleason	iPSA	PSA nadir	dt PSA (mo.)	PSA before FEC	PET/CT	Previous therpay
1	73	cT2 cN0	7	11,5	-	3	11,5	pos. (P)	-

2	59	cT2 cN0	6	9,1	-	7	9,1	pos. (P)	-

3	64	cT3 cN0	n.a.	731	-	n.a.	36	pos. (P, Bo., Bl)	AHT

4	77	cT3a cN0	7	3,4	-	n.a.	3,4	pos. (P)	AHT

5	71	cT3b cN0	8	2,5	-	n.a.	2,5	pos. (P, Ln)	AHT

6	77	cT3b cN0	7	10,4	-	n.a.	10,4	pos. (P)	AHT

7	74	cT3b cN0	7	27,5	-	n.a.	0,2	pos. (P)	AHT

8	60	cT2 cN0	7	13	-	3	13	pos. (Ln)	TUR-P, Seed

9	65	cT2 cN0	9	26,9	n.a.	n.a.	-	pos. (lLn)	RT P

10	63	pT1c cN0	6	4,71	3,49	n.a.	2,2	pos. (P)	RP, AHT

11	67	pT2a cN0	7	4,78	0,69	3	1,92	pos. (Ln)	RP

12	58	pT2b pN0	7	8,24	< 0,04	1	0,51	pos. (Ln)	RP, LAD

13	68	pT2c pN0	9	12,1	0,23	7	0,48	pos. (P, Ln)	RP, LAD

14	66	pT2c cN0	5	3,35	0,1	10	2,21	pos. (Ln)	RP, LAD

15	65	pT2c cNx	7	7,45	0,13	<3	1,19	pos. (P, Ln)	RP, AHT

16	71	pT3a cN0	10	n.a.	n.a.	n.a.	6,5	pos. (P, Ln)	TUR-P, AHT

17	71	pT3a pN1	7	n.a.	n.a.	n.a.	4,8	pos. (Ln)	RP, LAD, AHT

18	71	pT3a pN0	5	7,76	0,03	6	1,25	pos. (P, Ln)	RP, LAD, RT P

19	69	pT3a cN0	7	15,4	0,18	6	0,65	neg.	RP

20	71	pT3a pN0	6	n.a.	0,38	> 12	0,75	neg.	RP, LAD

21	65	pT3b pN1	7	14	0,89	4	1,69	pos. (Ln)	RP, LAD, AHT

22	70	pT3b pN0	7	43	0,62	3	1,89	pos. (P, Ln)	RP, LAD, AHT

23	69	pT3b pN0	7	14,3	0,17	2,5	0,42	pos. (Ln)	RP, LAD

24	55	pT3b pN0	6	15	0,35	n..a.	1,2	pos. (Ln)	RP, LAD

25	68	pT4 pN0	7	n.a.	n.a.	6,3	3	pos. (P, bone)	RP, LAD, AHT

26	74	pT3a pN0	5	7,76	0,03	< 3	2,87	pos. (Ln)	RP, LAD, RT P+Ln

Abbreviation: stage: initial tumor stage; iPSA: initial PSA value (ng/ml); PSA nadir: minimal PSA value after prostatectomy (ng/ml); dt PSA: PSA doubling in months; PET/CT: FEC-PET/CT result (P: FEC uptake in prostate gland or prostate bed; Ln: FEC uptake in lymph nodes; Bo: FEC uptake in bone; Bl: FEC uptake in bladder).

Previous therapy: Therapeutic modalities before PET/CT-planned irradiation. RT: radiotherapy (P: prostate; Ln: pelvic lymph nodes); AHT: anti-hormonal therapy; RP: radical prostatectomy; LAD: pelvic lymphadenectomy. n.a.: not available.

Previous therapy of recurrent cancer was radical prostatectomy with or without lymphadenectomy in 16/19. Two patients had transurethral resection with seed implantation or antihormonal therapy; one had radiotherapy of the prostate gland only. In primary cancer, 5/7 were treated with neoadjuvant and/or adjuvant antihormonal therapy.

### Imaging

Staging included physical examination with digital rectal palpation, complete laboratory tests, FEC-PET/CT, and MRI with endorectal coil (routinely used since 2007). No bone scintigraphy was required.

PET/CT studies were performed on a combined PET/CT scanner (Siemens Biograph 16) with radiation therapy equipment (Siemens Medical Solutions, Erlangen, Germany). All patients fasted for at least 4 hours before the 18F-fluoroeythylcholine (FEC) PET study. After FEC injection (350 - 500 MBq; 5 MBq/kg), a dual-time-point PET/CT scan was carried out in all patients. Early acquisition including the pelvis and lower abdomen started 2 minutes after tracer injection, before the tracer normally reaches the bladder. To significantly reduce bladder activity in the delayed scan, patients received 20 mg furosemide and were instructed to drink 1-1,5l of water for forced diuresis. After bladder voiding late scans started 60-90 minutes post injection. Imaging was done from skull base to the upper thigh. Static 3D PET data were acquired at 3 minutes per bed position. No dynamic acquisition was performed. Standard uptake values (SUV) are reported as SUVmax values. Region of interest (ROI) were ellipsoid volumes of interest with appropriate dimensions to only include the interesting structure.

The acquisition protocol included a full diagnostic CT scan native and with i.v. contrast. In addition to standard 5 mm slice thickness, 2 mm slices with 1 mm increments were reconstructed (2D OSEM iterative reconstruction algorithm) for diagnostic purposes and multi-planar-reformation.

PET/CT interpretation was performed by an experienced nuclear medicine physician/radiologist (AW). A multimodality computer platform (TrueD - Syngo Multimodality Workplace, Siemens Medical Solutions, Erlangen, Germany) was used for image review and interpretation.

Visual assessment of focal increased tracer uptake higher than the surrounding background was used as a criterion for malignancy. High focal uptake in the prostate and prostate region was considered to be primary tumor/recurrent disease. Focal increased uptake in the pelvic and retroperitoneal lymph nodes or in the skeleton were interpreted as metastatic disease. Mild tracer uptake in distal iliacal and inguinal lymph nodes occurred regularly and was considered as reactive. Care was taken to differentiate physiologic high choline uptake from sites with pathologic uptake.

### Radiation therapy

Treatment planning was done with Masterplan (Nucletron, The Netherlands) in case of 3D-conformal technique and KonRad or Prowess Panther DAO in case of IMRT planning. Patients were treated five times weekly with 1,8 (2,0) Gy/fraction up to a median dose of 75,6 Gy (range: 72 - 75.6 Gy) in primary cancer and 66,6 Gy (range 55.8 to 75.6 Gy) in recurrent disease. In low risk patients with primary disease, only the prostate gland without seminal vesicles were included in the clinical target volume. In intermediate and high risk patients with primary disease, the prostate gland and seminal vesicles were included up to a maximum dose of 66,6 Gy. In case of involvement of the seminal vesicles, the involved part of the seminal vesicle was carried to the maximum dose of 75,6 Gy. The planning target volume (PTV) included the CTV plus 8 -10 mm safety margins lateral, longitudinal and ventral, and 5 to 8 mm dorsal for a maximum dose of 70,2 Gy, or 3 - 5 mm dorsal from 70,2 to 75,6 Gy.

Pelvic lymph nodes were treated in case of FEC-PET/CT positive lymph nodes or a risk of lymph node involvement greater 20% according to *The Artificial Neural Networks in Prostate Cancer Project *(ANNs in CaP; http://www.prostatecalculator.org). In case of pelvic lymph node irradiation, all pelvic lymph nodes up to the level of L5/S1 were irradiated with a total dose of 45 Gy in 3D conformal irradiation or 50,4 Gy in IMRT with a boost to the FEC-PET/CT positive lymph nodes. In recurrent cancer, the dose to the prostatic bed was 60 Gy from 2006 to 2008 and thereafter 64 to 66,6 Gy. If a FEC PT/CT positive foci was detected in the prostatic bed, a boost dose was given up to 70,2 Gy or 75,6 Gy in one case of a large macroscopic nodule. Volumes of prior irradiation were excluded except for one case with a recurrence in seminal vesicles. The dose to 20% of the rectum (V20) was kept to a maximum of 70 Gy. Weekly portal imaging was done in the case of 3D conformal irradiation or with megavoltage cone beam CT (CBCT) in the case of IMRT, with daily CBCT's during the first week and thereafter once weekly.

Linear accelerators with 6 and 10 MV photons were used equipped with electronic portal imaging (Siemens Oncor) or Megavoltage Cone Beam CT (Siemens Artiste).

### Follow up

Clinical outcome was determined from regular follow-up visits 6 to 8 weeks after the end of radiotherapy and thereafter every six to twelve months and/or a questionnaire or telephone consultations of urologists assigned with the primary care of the patients. The median follow up time was 28 months.

### Statistical analysis

Outcomes were defined from the start of irradiation. Kaplan-Meier curves were used to estimate overall, biochemical relapse free and distant disease free survival. R-square given is a correlation coeefficient. All statistical analyses were done with GraphPad Prism (version 5.0c; GraphPad Software Inc.).

## Results

### FEC-PET/CT

**Figure 1:**
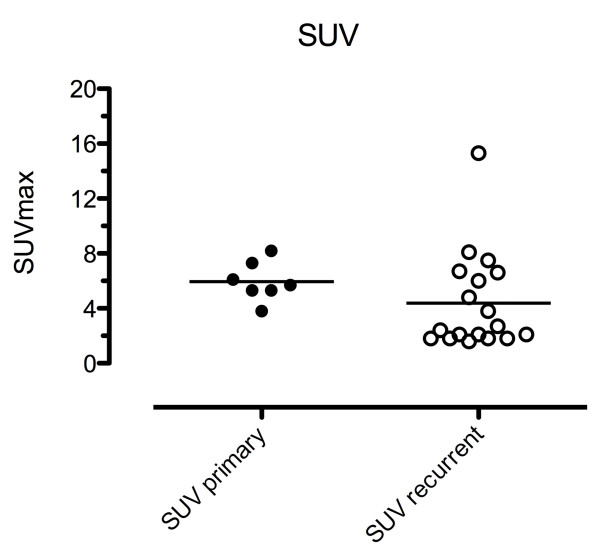
**SUV_max _for primary and recurrent prostate cancer**. The maximum standardized uptake value (SUV_max_) is given for primary and recurrent prostate cancer receiving a Choline-PET/CT for diagnosis and radiotherapy treatment planning. The mean SUV_max _for primary and recurrent cancer are shown. The difference is not significant (p = 0.089).

The PET/CT-studies were positive in 24/26 cases. In primary cancer, one patient had bone metastases and bladder infiltration, and one had FEC-uptake in the prostate gland and pelvic lymph nodes. In Figure 1, maximum standardized uptake values (SUV_max_) are shown. The mean SUV_max _in primary cancer was 5,97 (range: 3.8 to 8.2) in the prostate gland and 3,2 in pelvic lymph nodes. Patients with recurrent cancer had a mean SUV_max _of 4,38 (range: 1,6 to 15,3). Two patients had negative PET/CT scans. Both had PSA values at time of FEC-PET/CT below 1 ng/ml (0,65 and 0,75 ng/ml). FEC uptake was found in recurrent tumours in the prostatic bed in 4 cases. FEC uptake in pelvic lymph nodes was found in the majority of cases in external iliac nodes (7/20; 35%), within the fossa obturatoria (4/20; 20%), and common iliac nodes (3/20; 15%). Two cases with presacral nodes were found. On contrast-enhanced CT the foci correlated with lymph nodes.

The median PSA at the time of PET/CT was 10.4 ng/ml(range: 0.2 to 115 ng/ml) for primary cancer and 1.9 ng/ml (range: 0.42 to 65 ng/ml) in recurrent cancer.

**Figure 2:**
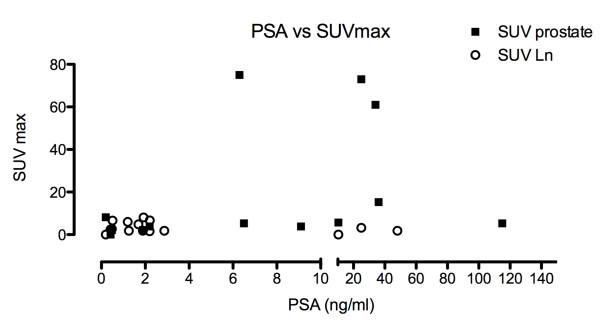
**SUV_max _and PSA**. The maximum standardized uptake value (SUV_max_) is shown as a function of PSA values (ng/ml) at the time of FEC-PET/CT scanning. Open symbols denote SUV_max _values of lymph nodes, closed squares those of the prostatic gland or fossa. No correlation was found (R^2 ^for combined data of prostate and lymph nodes was 0,02224; p = 0.45).

No correlation was found between PSA at the time of PET/CT and SUVmax of the prostate gland or fossa or lymph nodes. The R square for combined data of SUV_max _prostate and lymph nodes was 0,02224 (p = 0.45), as shown in Figure 2.

### Survival

**Figure 3:**
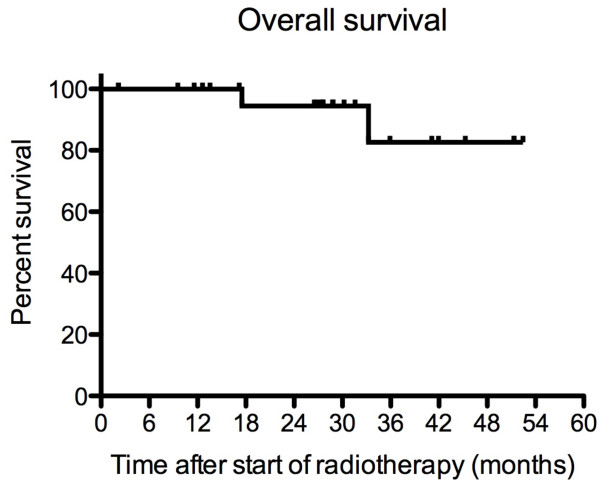
**Overall survival**. The overall survival rates are shown for all patients (n = 26). The 2 and 3 year survival rates were 94.4% and 82.6%.

**Figure 4:**
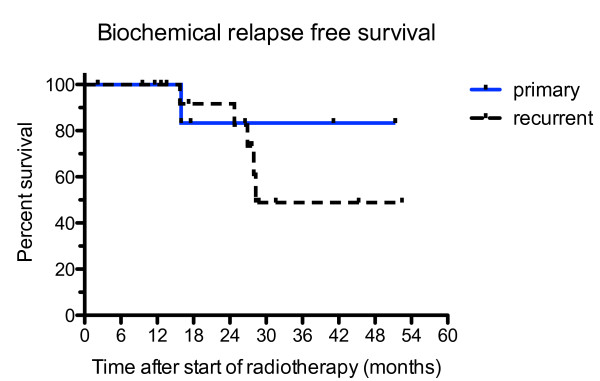
**Biochemical relapse free survival**. The biochemical relapse free survival (BRFS) rates are shown for recurrent (n = 17; dashed line) and primary prostate cancer (n = 7; blue solid line). The 2 and 3 year BRFS rates are 83.3% and 83.3% for primary, and 82.5% and 48.9% for recurrent tumours. The median survival time for recurrent tumors is 28.3 months and not reached for primary cancers. The difference between primary and recurrent cancers is not significant (p = 0.6).

**Figure 5:**
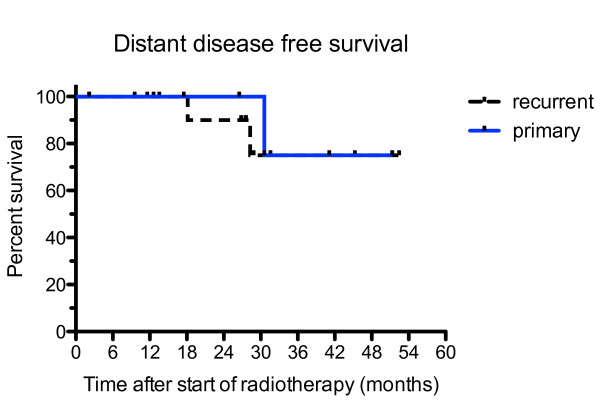
**distant disease free survival**. The distant disease free survival (DDFS) rates are shown for recurrent (n = 15; blue solid line) and primary prostate cancer (n = 7; dashed line). The 2 and 3 year DDFS rates are 100% and 75% for primary, and 90% and 75% for recurrent tumours. The difference between primary and recurrent cancers is not significant (p = 0.51).

Overall survival of all PET/CT-planned patients is depicted in Figure 3. At 28 months (median follow up time), the survival rate is 94%. In Figure 4, the biochemical relapse free survival (BRFS) is given. For primary tumours, the BRFS is 83% at 28 months, whereas, it is 49% for recurrent tumours. The median survival time for recurrent tumours is 28.3 months and not reached for primary cancer. In Figure 5 the distant disease free survival (DDFS) is shown. Patients with primary tumours have 100% DDFS rate at 28 months and 75% for patients with recurrent disease.

**Figure 6:**
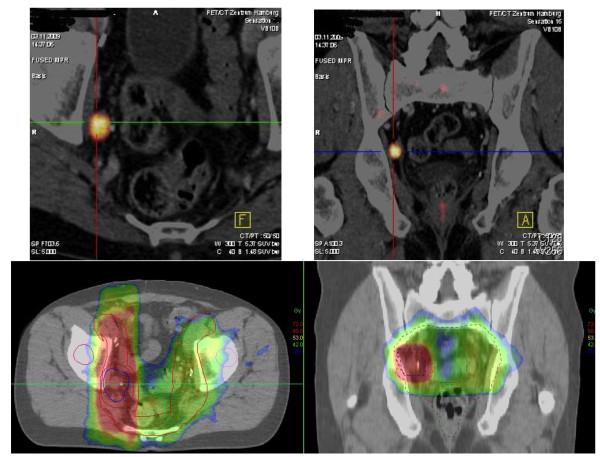
**Dose distribution of FEC-PET/CT planned IMRT**. An example of F18-fluoroethylcholine PET/CT-planned IMRT is shown (patient number 24, Table 1). PET/CT- fused images (upper part) showed a single positive lymph node in the right fossa obturatoria with an SUV _max _of 6,0 and an maximum diameter of 1,6 cm. The patient received IMRT irradiation of the pelvic lymph nodes to 45 Gy (five weekly fractions of 1,8 Gy) with a total boost dose of 66,6 Gy applied to the lymph node metastases (lower part of figure; colour wash images of transversal and coronal plane are depicted. Green: 42 Gy; red to orange: 60 to 72 Gy).

An example of the dose distribution of FEC-PET/CT-planned IMRT radiotherapy is given in Figure 6 (patient number 24, table 1).

### Patterns of relapse

A relapse in multiple lymph nodes and bone metastases occurred in one patient after 15.7 and 41.9 months. In another patient, a paraaortic lymph node relapse outside the initial radiotherapy portals was observed 28.3 months after PET/CT-planned radiotherapy. He declined chemotherapy of his hormone refractory cancer and chose instead a FEC-PET/CT-planned IMRT of the paraaortic lymph nodes with dose-escalated boost to the lymph node metastases.

One patient, who initially had radiotherapy of the prostate gland and antihormonal therapy, had his first relapse 3 years later in multiple pelvic and paraaortic and lymph nodes. As he experienced a further progress in pelvic lymph nodes he was referred to radiotherapy. Systemic therapy was difficult because of multiple co-morbidities. A PET/CT-planned 3D conformal radiotherapy of the pelvic lymph nodes was performed with boost to the metastatic lymph node of 60 Gy. Bone metastases were detected 18.3 months after PET/CT planned radiotherapy. The patient had an infield, symptomatic pelvic lymph node recurrence 26.8 months after PET/CT-planned radiotherapy and was offered IMRT reirradiation to alleviate pain.

Two patients died during the follow-up period. Death occurred in one patient at 33.3 months because of bone metastases. Another patient died unexpectedly of unknown cause and without signs of prostate cancer at 17.5 months.

### Toxicity

Acute toxicity was minimal or mild in 22/26 (85%) patients. Moderate side effects of the rectum or bladder occurred in 4/26 (15%). No or mild late side effects were observed in the majority of patients (84%). Two patients had moderate rectal problems (grade 2, CTC) and one patient had moderate fatigue. In one patient symptomatic bone pain requiring analgesics developed 25 months after the end of treatment. Signal alterations of the sacrum with oedema but no evidence of bone metastases were found in an MRI. Complete relief could be achieved within 7 months. No evidence of disease was found 52.4 months after end of treatment with a PSA of 0.

One case with a severe grade 4 late effect of the bladder was observed. The patient initially had a TUR of the prostate gland and seed implantation in curative intention. He experienced a recurrence in a seminal vesicle and in iliac lymph nodes 54 months after initial treatment. The patient was extensively informed about the potential risks of reiiradiation after declining alternative tretament options e.g. docetaxel chemotherapy. Based on this individual treatment decision, he received PET/CT-planned IMRT to the prostate gland, seminal vesicles and pelvic lymph nodes of 45 Gy with a boost to the PET/CT positive seminal vesicle and lymph nodes of 55.8 Gy at 1.8 Gy per fraction. Severe bladder shrinkage made a bladder removal necessary with construction of a transversum conduit 2 years after PET/CT IMRT. He is alive and without evidence of disease 28.8 months after PET/CT IMRT.

## Discussion

In this single institutional experience, 26 patients with mainly intermediate to high risk primary or recurrent prostate cancer received FEC - PET/CT planned radiotherapy with escalated boost doses to PET/CT positive lymph node sites. Doses to lymph nodes of up to 66,6 Gy were well tolerated. Local control rates after a median follow up time of 28 months are encouraging with only two documented infield recurrences.

F18-fluoroethylcholine/11C-choline have been developed as imaging probes in PET imaging [7]. It might be helpful in target volume definition in radiotherapy especially for irradiation of nodal sites in the absence of reliable conventional imaging modalities as MRI and CT. The accuracy of PET/CT in detecting lymph node metastases in patients with a PSA relapse has only been assessed in a few studies to date. In one prospective study [8], 22 of 36 patients had a PSA relapse after curative treatment for prostate cancer and were re-staged with 11C- choline PET. Five of these patients (four after radical prostatectomy, one after radiotherapy) showed increased uptake of choline in pelvic lymph nodes. After lymphadenectomy, all five of these patients were found to have metastatic nodal disease. The same group examined in a prospective study 67 consecutive patients with histological proven prostate cancer with 11C-choline PET [9]. Of these, 43 patients had pelvic lymphadenectomy and 15/43 patients had histologically proven lymph node metastases. 11C-Choline PET was true-positive in 12 patients with uptake of 11C-choline in pelvic lymph nodes and false-negative in 3 patients. A total of 27 metastatic lymph nodes were identified after pelvic lymphadenectomy. 11C-Choline PET identified 19 of 27 (70%) of these metastatic nodes. The sensitivity of 11C-PET/CT was 80%, the specificity 96%, and accuracy 93%.

It is unclear at which PSA level choline PET/CT might have an impact on treatment decisions [10,11]. Cimitan et al. [5] studied 100 consecutive patients with PET/CT because of biochemical relapse of prostate cancer. The majority of negative PET/CT scans (41/46) were observed in patients with a post-treatment serum PSA <4 ng/ml, and most true positive PET/CT scans (43/53) were observed in patients with serum PSA >4 ng/ml. They suggested limiting PET/CT to selected patients with higher PSA levels and/or poorly differentiated prostate carcinoma (Gleason score 7 or higher). In the current study, both patients with a negative FEC-PET/CT had PSA values below 1 ng/ml at the time of PET/CT.

We did not use FEC-PET/CT-directed dose escalation on intraprostatic lesions. Niyazi et al. [12] employed a mathematical model assuming various PET detection rates and alpha-beta values to estimate the effect of dose escalation on intraprostatic lesions. The model was based on several fundamental assumptions (uniform clonogenic cell density, no interaction between adjacent tumor cells, no sub-volume effects and a uniform radio-sensitivity of all tumor cells). No time factors were considered. Outcome was highly variable depending on the inital assumptions. The authors' conclusions were skeptical about the possibilty of achieving clinically meaningful increases in local tumour control rates with this approach. It is unclear whether this skepticism also holds true for dose escalation to lymph nodes as was performed in the current study. The therapeutic benefit might be higher, because dose escalation is usually restricted to the prostate gland but not to lymph nodes due to the inaccuracy of conventional imaging modalities and concerns about bowel toxicity. Thus, total doses to lymph nodes are generally limited to below 60 Gy. In the current study we could demonstrate that total doses to metastatic lymph nodes of up to 66,6 Gy applied with IMRT and IGRT are safe and well tolerated. Acute and late toxicity to small bowel was not increased compared to standard approaches with lower doses to pelvic lymph nodes. The observed local and regional control rates are encouraging. Thus, our patients seemed to benefit from increased locoregional control rates without increased normal tissue complication rates.

Though molecular imaging with choline PET/CT is promising, careful interpretation of PET/CT findings and consideration of clinical data is necessary in decision-making. Spatial resolution of the current PET/CT scanners is still limited to about 5 - 8 mm. In addition, interpretation of PET/CT data may be difficult in several circumstances. Examples are a possibly decreased sensitivity of choline-PET in androgen-deprived patients [13] and inflammatory tissue changes resulting in an increased uptake of choline imitating cancer growth [14].

## Conclusions

F18-fluoroethylcholine-PET/CT could be helpful in dose escalation in prostate cancer allowing boost doses > 60 Gy to metastatic lymph nodal regions if PET/CT-planned intensity modulated and image guided radiotherapy is used. Thus, there might be still a curative chance for selected patients with metastatic lymph nodes or recurrent disease.

## Competing interests

The authors declare that they have no competing interests.

## Authors' contributions

FW, CP, JD were responsible for treatment decisions, dose prescription and target volume delineation. Data analysis was performed by FW. MK was responsible for treatment planning. AW performed the PET/CT studies and interpretation of results. All authors read and approved the final manuscript.
